# Effect of postoperative utilization of somatostatin on clinical outcome after definitive surgery for duodenal fistula

**DOI:** 10.1186/s40001-023-00988-w

**Published:** 2023-02-03

**Authors:** Weiliang Tian, Risheng Zhao, Shikun Luo, Xi Xu, Guoping Zhao, Zheng Yao

**Affiliations:** 1 Department of General Surgery, Jinling Hospital, Nanjing, Jiangsu China; 2 Department of General Surgery, Jiangning Hospital, Hushan Road No.169, Nanjing, Jiangsu China

**Keywords:** Duodenal fistula, Recurrence, Surgery, Outcomes, High-output

## Abstract

**Purpose:**

To evaluate the effect of postoperative utilization of somatostatin after definitive surgery for duodenal fistula (DF) in preventing a recurrence.

**Methods:**

Patients with definitive surgery for DF between January 2010 and December 2021 were categorized based on the utilization of somatostatin or not after the surgery. Patients in the Somatostatin group were matched to those in the Non-somatostatin group using propensity scores matching (PSM), so as to evaluate the effect of postoperative use of somatostatin by comparing the two groups.

**Results:**

A total of 154 patients were divided into the in the Somatostatin group (84) and the Non-somatostatin group (70). Forty-three patients (27.9%) exhibited a recurrent fistula, with which the postoperative use of somatostatin was not associated (19 [22.6%] in the Somatostatin group and 24 (34.3%) in the Non-somatostatin group; unadjusted OR 0.56; 95% CI 0.28–1.14; *P* = 0.11). However, the postoperative usage of somatostatin served as a protective factor for developing into high-output recurrent fistula (eight (13.3%) in the Somatostatin group and 15 (25%) in the Non-somatostatin group; adjusted OR 0.39; 95% CI 0.15–0.93; *P* = 0.04). After PSM, the recurrent fistula occurred in 29.2% subjects (35/120). The postoperative usage of somatostatin was not associated with recurrent fistula (13 in PSM Somatostatin group vs. 22 in PSM Non-somatostatin group; unadjusted OR 0.48; 95% CI 0.21–1.07; *P* = 0.07), while its postoperative usage decreased the incidence of recurrent high-output fistula (5/60 in the PSM Somatostatin group, compared with 13/60 in the PSM Non-somatostatin group; adjusted OR 0.30; 95% CI 0.09–0.95).

**Conclusion:**

Postoperative use of somatostatin could effectively reduce the incidence of recurrent high-output fistula, without association with overall incidence of postoperative recurrent fistula.

## Introduction

Duodenal fistula (DF) appears as a complication of abdomen and kidney surgeries, severe pancreatitis, and inflammatory bowel disease (IBD) [1], with a mortality from 7 to 54% [1–5]. Benefiting from the good nutritional status and effective source control, DF can be closed spontaneously in most cases. However, spontaneous closure could not be achieved in a portion of patients for long time [3, 4]. Pointing to this conundrum, the surgical closure is implemented by some experienced specialists [5, 6]. However, the surgical closure without a regular procedure is often accompanied by great challenges, followed by a high risk of recurrent fistula [5, 6].

Somatostatin as a critical hormone secreted by the gastrointestinal tract plays a primary role in gastrointestinal function [7]. Among those various effects of somatostatin, the inhibition of digestive juice secretion [8], and reduction of the pressure in the digestive tract [9] are currently exploited to prevent the occurrence of postoperative pancreatic fistula after pancreaticoduodenectomy, which achieves good outcomes in clinics, as reported in a majority of literature [10–13]. Theoretically, the postoperative use of somatostatin could also suppress the secretion of duodenal fluid involving a large amount of pancreatic juice, and thus achieve a lower recurrent DF.

The objective of this study is to validate the hypothesis above and evaluate the effect of postoperative use of somatostatin on the recurrent DF after the definitive surgery.

## Materials and methods

This retrospective cohort study was conducted at two regional large enterocutaneous fistula (ECF) centers, where hundreds of patients with refractory ECF are treated every year. All investigations were performed in accordance with the Declaration of Helsinki ethical standards.

### Patients

Patients with definitive surgery for DF between January 2010 in December 2021 were categorized according to whether somatostatin was used after the surgery. The characteristics were compared between the two groups. Patients younger than 18 years of age, or suspected with tumor recurrence that may affect the observation results were excluded.

### Follow-up

Patients were followed up to discharge. The primary outcome for investigation was the incidence of recurrent fistula, the secondary for length of stay (LOS) after surgery.

### Preoperative management

The treatment of DF followed the SOWATS treatment guideline, which consisted of six dimensions: sepsis, optimization of nutritional state, wound care, anatomy (of the fistula), timing of definitive surgery, and surgical strategy [14]. Nasojejunal feeding was adopted for preoperative enteral nutrition. In addition, in patients with high-output fistula, duodenal leakage fluid was collected and transfused via nasojejunal tube.

The definitive surgery would not be considered until the following conclusive criteria could be met. Firstly, C-reactive protein (CRP), white blood cell (WBC), and procalcitonin (PCT) are maintained normal for more than one month. Secondly, body mass index (BMI) >  = 18.0 with normal physical strength. Thirdly, hemoglobin >  = 100 g/L, and albumin >  = 30 g/L. Lastly, the interval exceeds 4 months after the first-time discharge from our institution.

### Surgical strategy

The definitive surgery was performed by a chief surgeon, Dr. Yunzhao Zhao, MD. & PhD., which can be described as: fistula can be identified and repaired or duodenojejunostomy can be performed during the surgery. The repair was routinely implemented. A Roux-en-Y anastomosis of duodenum and jejunum would be carried out for the fistula with a defect exceeding one-third of the duodenal circumference. During the process, the jejunum was transected at 20 cm from the Treitz ligament, and the distal end was anastomosed with the duodenum manually. The proximal end was anastomosed with the small intestine at 40 cm from the duodenal–jejunal anastomosis. A double-lumen irrigation-suction tube [15] was placed near the duodenal–jejunal anastomosis, in case of postoperative recurrent fistula to achieve postoperative irrigation and drainage.

In addition, hernia repair was also implemented in the definitive surgery to patients with planned abdominal hernia after open abdomen treatment. In the process, component separation technology was applied with on lay mesh repair, and a Cook Biodesign advanced tissue repair device (Cook Medical Inc., Bloomington, IN, USA) was employed. Negative pressure drainage was used under all incisions. For the postoperative treatment, RBC and human serum Alb were taken to maintain patients with hemoglobin (Hb) > 100 g/L and/or albumin (Alb) > 30 g/L, respectively.

### Postoperative management

A high incidence of postoperative complications will appear after surgical management of DF [5, 6]. Inspired by the utilization of somatostatin after pancreatoduodenectomy, we attempted to prevent recurrence of fistula with postoperative use of somatostatin after duodenal fistula surgery. Somatostatin was uniformly pumped at a dose of 250 ug/h from the first day after surgery, and stopped on the seventh day after surgery no matter if the recurrent fistula occurred. However, the above strategy is not encouraged by medical insurance policy in our country on somatostatin which was formulated according to the instructions. With the strict medical insurance policy, the use of somatostatin has been gradually stopped since 2015.

### Assessment of the recurrent fistula

The assessment of postoperative recurrent intestinal fistula included the diagnosis and output of the fistula. Patients received an upper gastrointestinal contrast (UGC) within 7 to 10 days after surgery, which served as the main base of diagnosis. The fistula with leakage more than 500 mL/day [16] lasting for more than three consecutive days after definitive surgery was considered as high-output postoperative fistula in the present study.

### Data collection and statistical analysis

Demographic data (gender, age, BMI), preoperative laboratory examination results and characteristics of fistula were recorded within one week before surgery. All statistical analyses were carried out using the statistical package for social science (SPSS) version 26.0 for Windows (IBM, Analytics, Armonk, NY). The Student’s t-test and Mann–Whitney U test were employed for continuous variables and the Fisher’s exact test for categorical variables. Postoperative outcomes across groups were compared using the Kaplan–Meier estimator and the log-rank test. Logistic regression and Cox regression were performed to assess the correlations of usage of somatostatin with other clinical indices. Patients with postoperative somatostatin were matched to those without postoperative somatostatin using 1:1 propensity score-matching with a tolerance level of 0.02, so as to reduce the impact of treatment-related bias on the practice of estimating the treatment effects with observational data. A *P*-value of < 0.05 was deemed statistically significant.

## Results

### Demographic and clinical characteristics

**Fig. 1:**
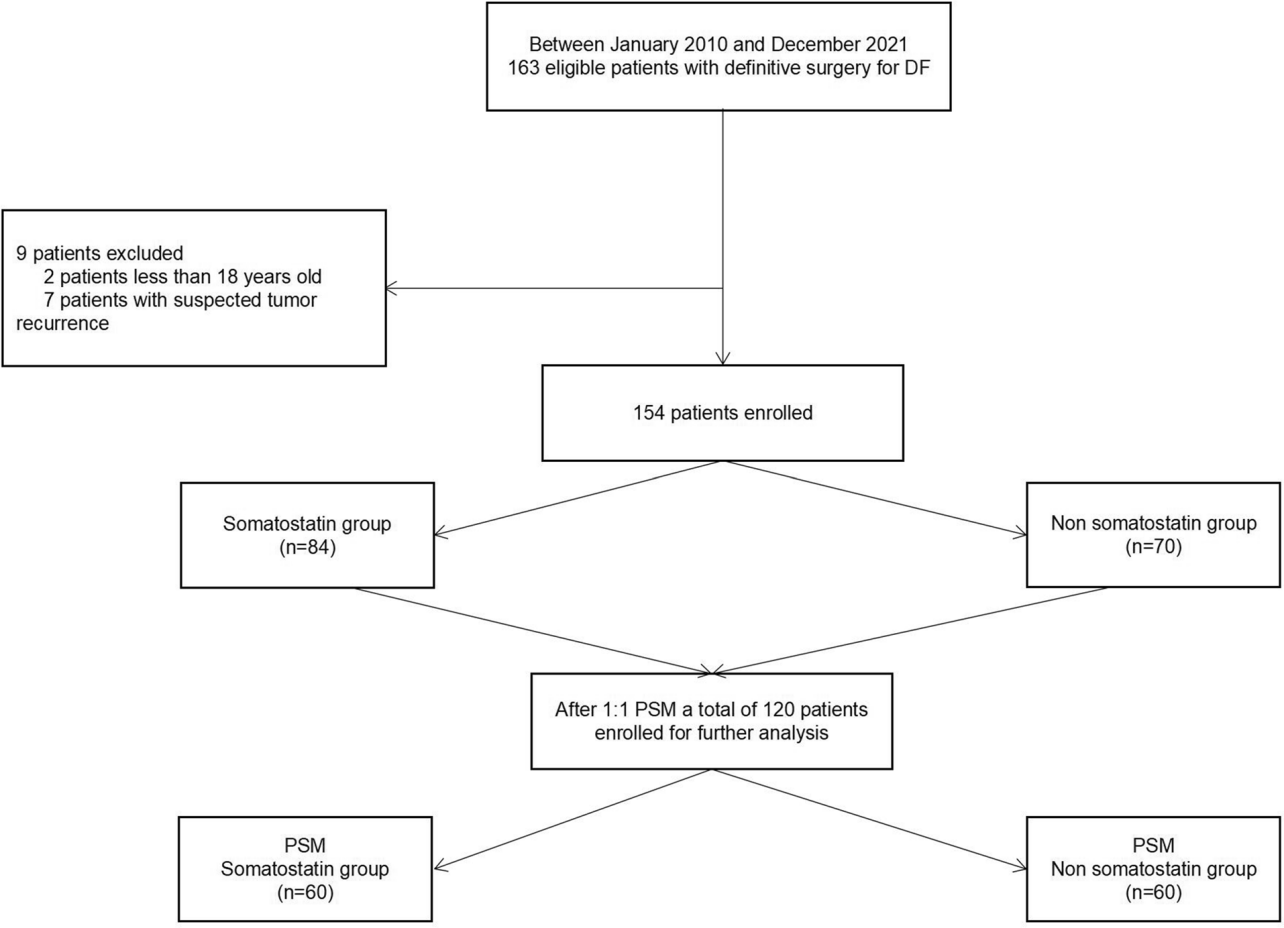
The patients and grouping

One hundred and sixty-three patients receiving a definitive surgery for DF were initially selected, among which nine were excluded. Thus, 154 eligible patients were enrolled in the study, with the median age of 46 years (interquartile range, IQR 33–60 years), 91 (59.1%) males. Eighty-four of the 154 patients were divided in the Somatostatin group and the rest 70 in the Non-somatostatin group (Fig. 1). 120 patients were enrolled (Fig. 1) after 1:1 PSM, with the median age of 44 years (interquartile range, IQR 33–58 years), and the proportion of males of 55.8% (67/120). The characteristics of the corresponding groups were comparable before and after PSM (Table 1).

**Table 1:** Characteristics of the patients

	Somatostatin group	Non-somatostatin group	*P*	PSM Somatostatin group	PSM Non-somatostatin group	*P*
Demographic data						
Gender, *n* (%)			0.83			0.85
Male	49 (58.3)	42 (60.0)		33 (55.0)	34 (56.7)	
Female	35 (41.7)	28 (40.0)		27 (45.0)	26 (43.3)	
Age, years, median (IQR)	45 (34–61)	46 (32–60)	0.86	45 (32–59)	42 (33–57)	0.96
BMI, kg/m^2^, median (IQR)	21.2 (19.8–22.1)	21 (19.8–22)	0.61	21.4 (19.8–22)	21 (19.7–22)	0.93
Characteristics of fistula						
Anatomy of fistula, *n* (%)			0.89			0.66
Stump	39 (46.4)	30 (42.9)		26 (43.3)	27 (45.0)	
Bulb or descending portion	40 (47.6)	35 (50)		32 (53.3)	29 (48.3)	
Pars horizontalis duodeni	5 (5.9)	5 (7.1)		2 (33.3)	4 (6.7)	
Etiology, *n* (%)			0.49			0.93
Upper gastrointestinal tract tumor	29 (34.5)	18 (25.7)		17 (28.3)	16 (16.7)	
Trauma	43 (51.2)	40 (57.1)		33 (55.0)	35 (58.3)	
Pancreatitis	12 (15.5)	12 (17.2)		10 (16.7)	9 (15.0)	
High output, *n* (%)	46 (54.8)	34 (48.6)	0.44	32 (53.3)	30 (50.0)	0.72
Combined with abdominal hernia, n (%)	38 (45.2)	33 (47.1)	0.81	28 (48.3)	26 (44.8)	0.71
Preoperative laboratory examination						
Preoperative hemoglobin, *n* (%)			0.98			0.85
> 100 g/L, and < 120 g/L	35 (41.7)	29 (41.4)		23 (38.3)	24 (40.0)	
≥ 120 g/L	49 (58.3)	41 (58.6)		37 (61.7)	36 (60.0)	
Preoperative albumin, n (%)			0.81			1.00
> 30 g/L, and < 35 g/L	38 (45.2)	33 (47.1)		28 (48.3)	28 (48.3)	
≥ 35 g/L	46 (54.8)	37 (52.9)		32 (53.3)	32 (53.3)	
Glutamic pyruvic transaminase, U/L (IQR)	31 (21–47)	31 (24–46)	0.72			
Total bilirubin, mmol/L (IQR)	20 (12–27)	21 (12–28)	0.71			
Creatinine, mmol/L (IQR)	58 (51–77)	61 (51–79)	0.67			
Intraoperative characteristics						
Blood loss, *n* (%)			0.79			1.00
< 1000 mL	45 (53.6)	39 (55.7)		33 (55.0)	33 (55.0)	
≥ 1000 mL	39 (4.4)	31 (44.3)		27 (45.0)	27 (45.0)	
Duration of definitive surgery, n (%)			0.42			0.71
< 4 h	51 (60.7)	38 (54.3)		32 (53.3)	34 (56.7)	
≥ 4 h	33 (39.3)	32 (45.7)		28 (48.3)	26 (43.3)	
Surgical procedure, *n* (%)			0.64			0.85
Suture	57 (67.9)	45 (64.3)		40 (66.7)	41 (68.3)	
Duodenojejunostomy Roux-en-Y	27 (32.1)	25 (28.6)		20 (33.3)	19 (31.7)	
Postoperative management						
Required blood transfusion within 48 h after definitive surgery			0.98			0.71
< 1000 mL	53 (63.1)	44 (62.9)		36 (43.3)	38 (63.3)	
≥ 1000 mL	31 (36.9)	26 (37.1)		24 (40.0)	22 (36.7)	
Required albumin transfusion within 48 h after definitive surgery			0.66			0.86
< 100 g	45 (53.6)	35 (50)		32 (53.3)	31 (51.7)	
≥ 100 g	39 (46.4)	35 (50)		28 (48.3)	29 (48.3)	
Comorbidity, No. (%)						
Hypertension	2 (2.3)	2 (2.9)	0.85	1 (16.7)	1 (16.7)	1.00
Diabetes mellitus	8 (9.5)	7 (10.0)	0.92	4 (66.7)	4 (66.7)	1.00

### Recurrent fistula

**Fig. 2:**
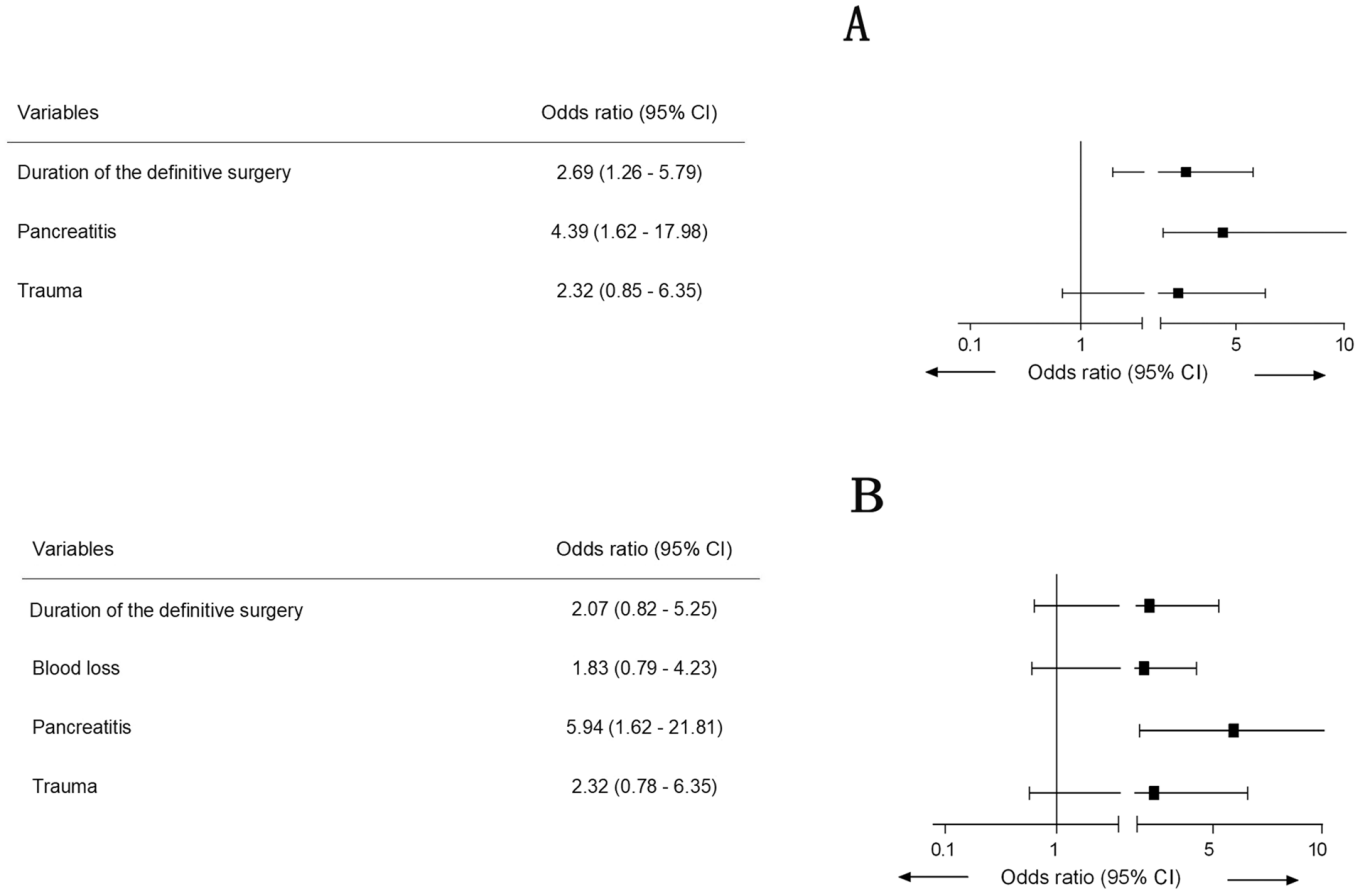
**A** Risk factors associated with recurrent fistula before PSM. **B** Risk factors associated with recurrent fistula after PSM

Before PSM, a recurrent fistula was reported in 43 (27.9%) of the 154 patients, including 19 (22.6%) in the Somatostatin group and 24 (34.3%) in the Non-somatostatin group. The incidence of recurrent fistula showed an insignificant decrease with the postoperative usage of somatostatin (unadjusted OR 0.56; 95% CI 0.28–1.14; *P* = 0.11), while the pancreatitis, as an etiology of duodenal fistula, was revealed associated with recurrent fistula before (adjusted OR 4.39; 95% CI 1.62–17.98; *P* = 0.009, Fig. 2A). A high-output recurrent fistula occurred in 23 (14.9%) patients, with 15 (25%) in the Non-somatostatin group and eight (13.3%) in the Somatostatin group. The postoperative usage of somatostatin could serve as a protective factor for developing into high-output recurrent fistula (adjusted OR 0.39; 95% CI 0.15–0.93; *P* = 0.04, Table 2).

**Table 2:** Logistics regression for postoperative high-output fistula before PSM

	OR	95% CI	*P*	OR	95% CI	*P*
Male	1.71	0.66–4.43	0.27			
Postoperative usage of somatostatin	0.39	0.53–0.97	0.04	0.39	0.15–0.93	0.04
Age	0.98	0.95–1.01	0.12			
BMI	0.98	0.73–1.29	0.86			
Anatomy of fistula						
Stump	Ref.					
Bulb or descending portion	1.58	0.82–3.05	0.18			
Pars horizontalis duodeni	1.38	0.36–5.21	0.64			
Etiology						
Upper gastrointestinal tract tumor	Ref.					
Trauma	1.82	0.55–5.99	0.33			
Pancreatitis	4.43	1.14–17.09	0.03	4.14	1.05–16.29	0.04
High output	0.82	0.34–2.00	0.67			
Combined with abdominal hernia	0.89	0.44–1.82	0.77			
Preoperative hemoglobin						
≥ 120 g/L	Ref.					
> 100 g/L, and < 120 g/L	1.13	0.46–2.79	0.79			
Preoperative albumin						
≥ 35 g/L	Ref.					
> 30 g/L, and < 35 g/L	2.04	0.81–5.00	0.13			
Blood loss						
< 1000 mL	Ref.					
≥ 1000 mL	1.09	0.45–2.64	0.86			
Glutamic pyruvic transaminase	1.01	0.77–1.21	0.74			
Total bilirubin	0.94	0.41–1.49	0.69			
Creatinine	0.99	0.56–1.28	0.61			
Duration of definitive surgery						
< 4 h	Ref.					
≥ 4 h	1.31	0.54–3.18	0.56			
Surgical procedure						
Suture	Ref.					
Duodenojejunostomy Roux-en-Y	1.13	0.44–2.87	0.79			
Required blood transfusion within 48 h after definitive surgery						
< 1000 mL	Ref.					
≥ 1000 mL	1.11	0.45–2.76	0.82			
Required albumin transfusion within 48 h after definitive surgery						
< 100 g	Ref.					
≥ 100 g	1.49	0.61–3.64	0.38			
Hypertension	2.39	0.24–24.04	0.46			
Diabetes mellitus	1.86	0.48–7.17	0.37			

After PSM, 35 (29.2%) of the 120 patients exhibited a recurrent fistula (13 in PSM Somatostatin group vs. 22 in PSM Non-somatostatin group), which was not influenced by the postoperative usage of somatostatin (unadjusted OR 0.48; 95% CI 0.21–1.07; *P* = 0.07), either. However, it was revealed that the pancreatitis was associated with recurrent fistula after PSM (adjusted OR 5.94; 95% CI 1.62–21.81; *P* = 0.007; Fig. 2B). The incidence of high-output recurrent fistula was 21.7% ([13/60] in the PSM Non-somatostatin group, as compared to 8.3% [5/60)] in the PSM Somatostatin group). The postoperative usage of somatostatin significantly inhibited recurrence of high-output fistula (adjusted OR 0.30; 95% CI 0.09–0.95; *P* = 0.04, Table 3).

**Table 3:** Logistics regression for postoperative high-output fistula after PSM

	OR	95% CI	*P*	OR	95% CI	*P*
Male	2.31	0.77–6.96	0.13			
Postoperative usage of somatostatin	0.33	0.11–0.99	0.04	0.30	0.09–0.95	0.04
Age	0.98	0.95–1.02	0.33			
BMI	1.02	0.73–1.43	0.91			
Anatomy of fistula						
Stump	Ref					
Bulb or descending portion	2.11	0.68–6.53	0.19			
Pars horizontalis duodeni	4.80	0.69–33.11	0.11			
Etiology						
Upper gastrointestinal tract tumor	Ref			Ref		
Trauma	1.53	0.38–6.06	0.55	1.49	0.37–6.05	0.57
Pancreatitis	4.62	0.99–21.34	0.05	5.05	1.05–24.36	0.04
High output	0.54	1.95–1.52	0.24			
Combined with abdominal hernia	0.80	0.36–1.81	0.59			
Preoperative hemoglobin						
≥ 120 g/L	Ref					
> 100 g/L, and < 120 g/L	1.29	0.47–3.56	0.62			
Preoperative albumin						
≥ 35 g/L	Ref					
> 30 g/L, and < 35 g/L	0.48	0.33–1.19	0.17			
Glutamic pyruvic transaminase						
Creatinine						
Blood loss						
< 1000 mL	Ref					
≥ 1000 mL	1.27	0.47–3.45	0.64			
Glutamic pyruvic transaminase	1.05	0.74–1.18	0.72			
Total bilirubin	0.96	0.52–1.39	0.49			
Creatinine	0.99	0.61—1.24	0.67			
Duration of definitive surgery						
< 4 h	Ref					
≥ 4 h	1.65	0.61—4.52	0.33			
Surgical procedure						
Suture	Ref					
Duodenojejunostomy Roux-en-Y	1.05	0.361—3.03	0.94			
Required blood transfusion within 48 h after definitive surgery						
< 1000 mL	Ref					
≥ 1000 mL	1.76	0.64—4.82	0.27			
Required albumin transfusion within 48 h after definitive surgery						
< 100 g	Ref					
≥ 100 g	1.91	0.69—5.33	0.22			
Hypertension	5.94	0.35—99.58	0.22			
Diabetes mellitus	2.00	0.37—10.79	0.42			

### LOS after definitive surgery

**Fig. 3:**
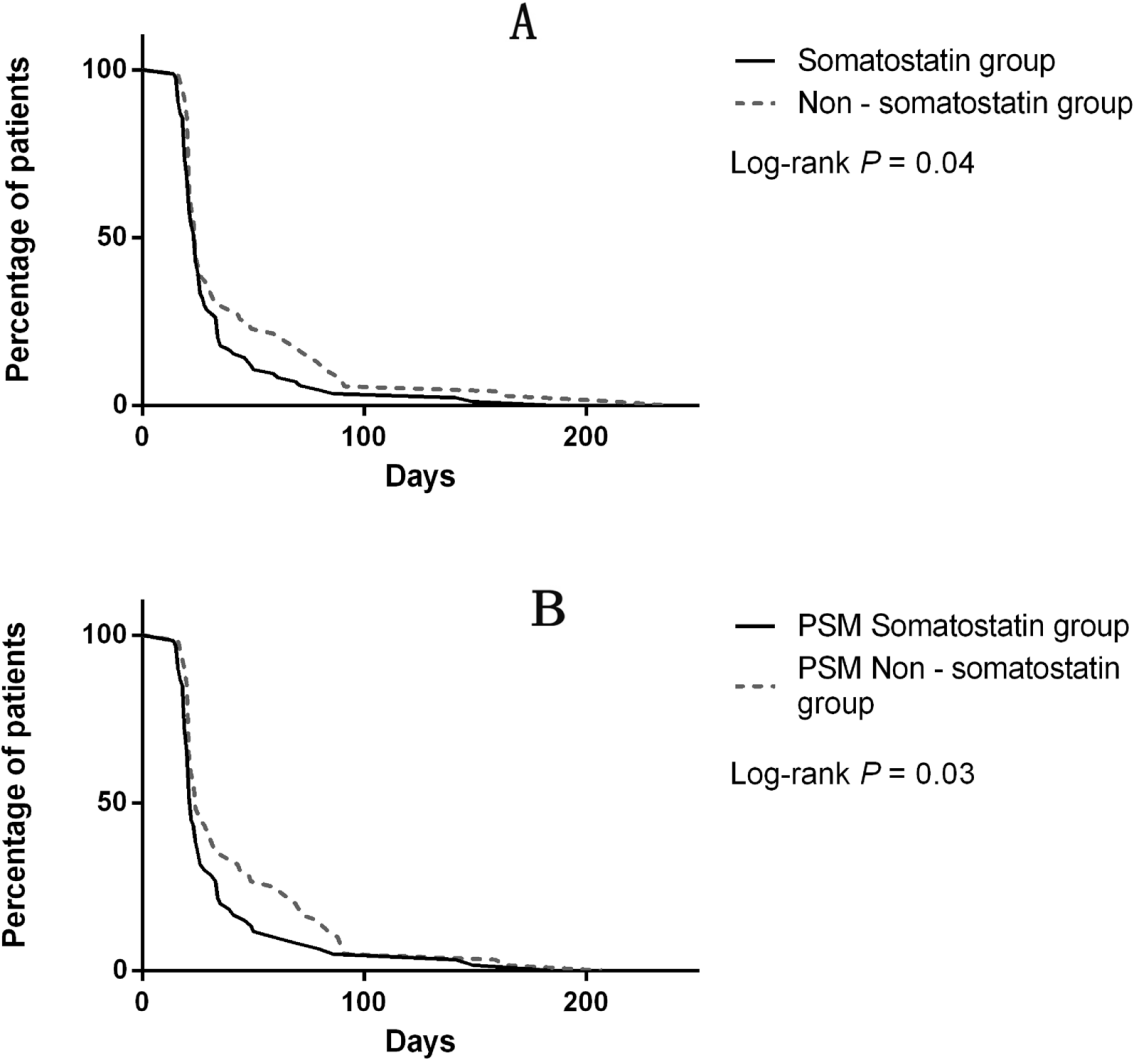
**A** Role of somatostatin in the early discharge before PSM. **B** Role of somatostatin in the early discharge after PSM

**Fig. 4:**
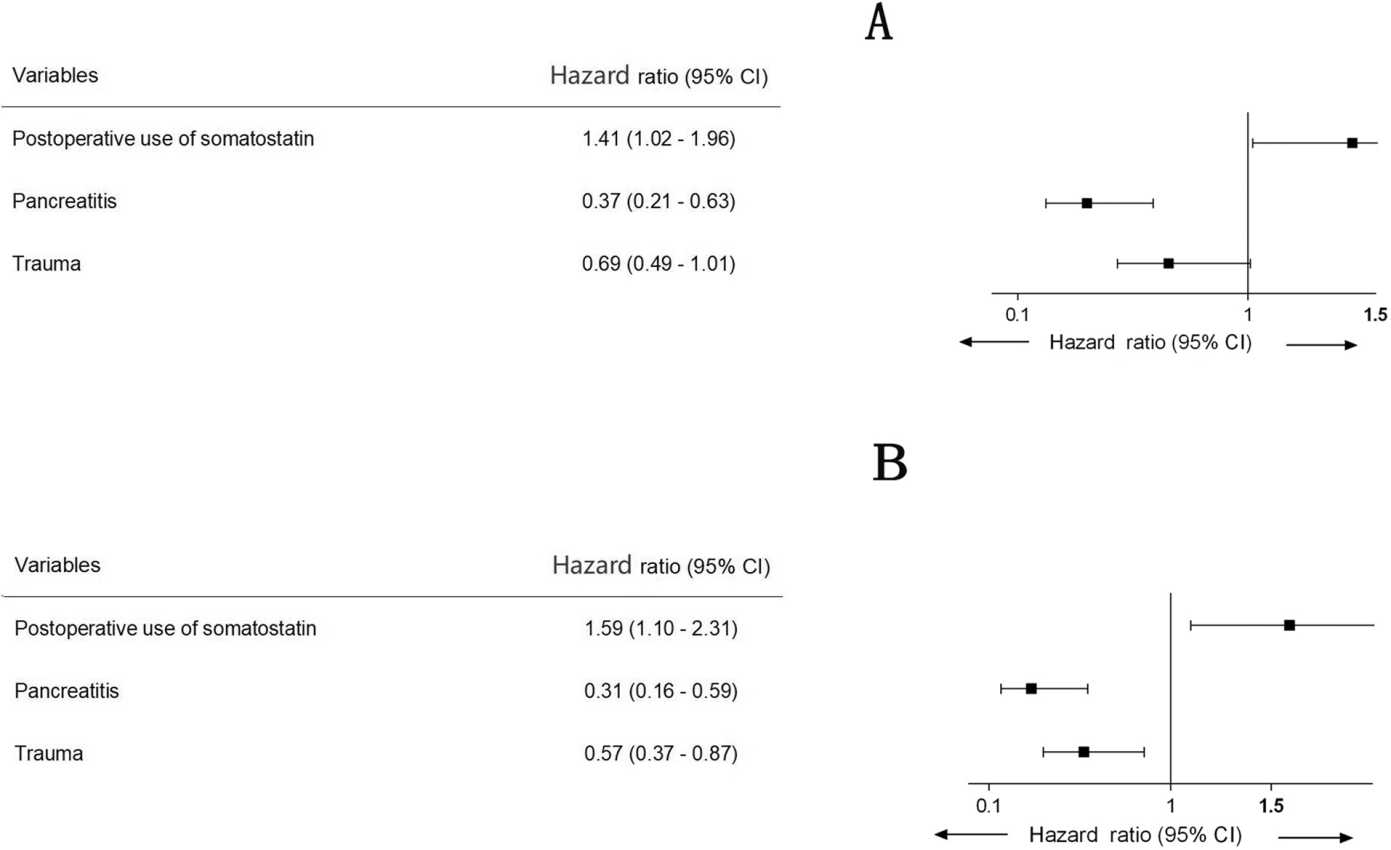
**A** Risk factors associated with earlier discharge before PSM. **B** Risk factors associated with earlier discharge after PSM

The postoperative death in the 154 patients was not detected. An additional definitive surgery was required in seven patients (three in the Somatostatin group, and four in the Non-somatostatin group) considering the unattainable spontaneous closure of the recurrent fistula. The overall mean LOS after definitive surgery of the 154 patients was 37.60 ± 2.92 days (32.71 ± 3.11 days in the Somatostatin group, and 43.47 ± 5.17 days in the Non-somatostatin group). The adjusted Cox regression revealed the conducive role of somatostatin in the early discharge (HR–1.41; 95% CI 1.02–1.96; *P* = 0.03, Fig. 3A and Fig. 4A).

After PSM, the mean LOS after definitive surgery was 39.24 ± 3.29 days, with 34.18 ± 4.21 days in PSM Somatostatin group, and 44.30 ± 5.00 days in PSM Non-somatostatin group. The usage of somatostatin was significantly associated with reduction of LOS (HR 1.59; 95% CI 1.10–2.31; *P* = 0.01, Fig. 3B and Fig. 4B) after correction of other relevant factors.

## Discussion

In the present study, the postoperative use of somatostatin was revealed to reduce the risk of high-output recurrent DF, under the comparable overall recurrent rate of DF between the two group. Somatostatin inhibits endocrine and exocrine functions of digestive tract [16]. It modulates gastrointestinal motility by delaying the late phase of gastric emptying, weakening gallbladder contraction, prolonging small intestinal transit time, decreasing the portal (and varicose) and digestive tract pressure [17]. With the advantage of those effects on digestive tract, the somatostatin has been attempted to use in the treatment of ECF. A review involving eight randomized controlled trial (RCT) showed that the use of somatostatin decreased the time to closure of fistulas compared to placebo [18]. However, in patients with DF which would be associated with high incidence of postoperative recurrent fistula, whether the postoperative somatostatin exerts an effect on improvement of outcomes is still unclear. Since the postoperative use of somatostatin was introduced by Klempa et al., to prevent the pancreatic fistula [19] in 1979 after pancreaticoenterostomy, the efficacy to reduce complications has been widely accepted by surgeons in Europe and Asia [10]. The mechanism has not been fully elaborated. However, an accepted view is that a postoperative use of the somatostatin reduces anastomotic pressure by inhibiting the pancreatic secretion [13]. The effect may mainly contribute to improving the prognosis after pancreaticoenterostomy. Duodenal fluid is primarily composed of bile and pancreatic juice. Theoretically, the correct use of somatostatin after definitive surgery for DF will reduce the secretion of duodenal fluid and the pressure in the duodenum, thus contributing to wound healing and positive outcomes. Another mechanism of postoperative using somatostatin to reduce the recurrent fistula might be that somatostatin, serving as a powerful inhibitor for severe inflammation [20], affects immune function and inflammation response and crosstalk [21]. Postoperative inflammation is a rather crucial promoting factor for recurrent fistula [22]. Major abdominal surgery always results in a cascading immune reaction, which plays an inhibitory role in the wound healing process and increase the risk of recurrent fistula [22]. In recent years, scholars have turned to focus on the relationship between postoperative inflammatory response and postoperative outcomes. Boelens et al. [22] have reported the association of the anastomotic fistula occurrence with postoperative inflammatory response in rectal tumor surgery. Similarly, in the research of Tian et al.[23], the decrease of postoperative inflammatory response was associated to the reduced occurrence of recurrent fistula after definitive surgery for ECF. Conclusively, the postoperative use of somatostatin contributing to the reduction of the recurrent fistula might be revealed by the decreased inflammatory reaction after surgery.

However, despite the inhibition of the digestive secretion, the decrease of splanchnic blood flow [24] and inhibition of angiogenesis [25] are also the impacts of somatostatin on the digestive system. These physiological effects might produce a negative influence on the wound healing of the duodenum. As local abundant blood supply is required to provide nutrients for the wound tissue, so as to promote the anastomosis to heal smoothly, while the anastomotic blood supply would directly lead to postoperative gastrointestinal fistula [15, 26]. From this perspective, somatostatin may instead aggravate the occurrence of recurrent fistula after definitive surgery for DF. McMillan et al. [27] even suppose that the postoperative use of the somatostatin should be cautious, because the reduction in pancreatic exocrine secretion owing to prophylactic somatostatin use is outweighed by the decreased splanchnic blood flow.

Therefore, the somatostatin may have brought a double-sided effect on postoperative recurrent fistula. Correspondingly, its influence on the recurrence of fistula may be relatively limited. However, when noting the recurrence of high-output DF, it could be comprehensively supposed that the positive effect of reducing digestive tract pressure on healing exceeded the negative effect of reducing blood supply on healing.

There were several limitations in our study. First, it was a retrospective study, in which selection bias was likely present. Second, the sample size was fairly limited, resulting in an elevated risk of bias. The conclusions were obtained according to the findings from this small cohort of 154 patients. Anyway, definitive surgery for DF is relatively rare, and our study was the first to explore the influence of the postoperative use of somatostatin on recurrent fistula. Additionally, in the further studies, more subjects would be enrolled. The third limitation was that the relatively large heterogeneity in etiology was selective, and DF was diagnosed after pancreatitis in a considerable number of patients, few literatures were focused on the DF following pancreatitis. However, in the present study, pancreatitis was associated with recurrence of DF and recurrent high-output fistula. It can be reasonable inferred that the surgical treatment in patients with pancreatitis might be more tough and pancreatitis as an etiology might produce a large impact on the outcomes of DF. As a result, the role of somatostatin in reduction of recurrent fistulas might be underestimated when a substantial number of DF following pancreatitis were enrolled. Additionally, the follow-up of patients was maintained to discharge. It can be inferred that the delayed recurrent fistula might not be recorded in few patients, which could induce a bias. However, the delayed fistula was relatively rare in gastrointestinal surgery, and a UGC was performed within 7 to 10 days after surgery in each patient. Most recurrent fistula could be detected in this imaging examination. Therefore, it could be inferred that the probability of delayed recurrent fistula was rather low.

## Conclusion

Postoperative use of somatostatin could reduce the incidence of recurrent high-output fistula, which was not associated with overall incidence of postoperative recurrent fistula.

## Data Availability

The datasets used and/or analyzed during the current study are available from the corresponding author on reasonable request.

## Abbreviations

BMI: Body mass index
CRP: C-reactive protein
DF: Duodenal fistula
DS: Definitive surgery
ECF: Enterocutaneous fistula
IBD: Inflammatory bowel disease
IQR: Interquartile range
LOS: Length of stay
PCT: Procalcitonin
PSM: Propensity scores matching
UGC: Upper gastrointestinal contrast
WBC: White blood cell

## References

[1] Yao Z, Ge Z, Xu X (2018). Prevalence of and risk factors for abdominal bleeding in patients with external Duodenal Fistula. Med Sci Monit.

[2] Milias K, Deligiannidis N, Papavramidis TS, Ioannidis K, Xiros N, Papavramidis S (2009). Biliogastric diversion for the management of high-output duodenal fistula: report of two cases and literature review. J Gastrointest Surg.

[3] Cozzaglio L, Giovenzana M, Biffi R (2016). Surgical management of duodenal stump fistula after elective gastrectomy for malignancy: an Italian retrospective multicenter study. Gastric Cancer.

[4] Zizzo M, Ugoletti L, Manzini L (2019). Management of duodenal stump fistula after gastrectomy for malignant disease: a systematic review of the literature [published correction appears in BMC Surg. BMC Surg.

[5] Rossi JA, Sollenberger LL, Rege RV, Glenn J, Joehl RJ (1986). External duodenal fistula. Causes, complications, and treatment. Arch Surg.

[6] Chander J, Lal P, Ramteke VK (2004). Rectus abdominis muscle flap for high-output duodenal fistula: novel technique. World J Surg.

[7] Martin-Grace J, Tamagno G (2015). Somatostatin analogs in the medical management of occult bleeding of the lower digestive tract. Gastroenterol Res Pract.

[8] Tulassay Z (1998). Somatostatin and the gastrointestinal tract. Scand J Gastroenterol Suppl.

[9] Nellgård P, Bojö L, Cassuto J (1995). Importance of vasoactive intestinal peptide and somatostatin for fluid losses in small-bowel obstruction. Scand J Gastroenterol.

[10] Cao Z, Qiu J, Guo J (2021). A randomised, multicentre trial of somatostatin to prevent clinically relevant postoperative pancreatic fistula in intermediate-risk patients after pancreaticoduodenectomy. J Gastroenterol.

[11] Callery MP, Pratt WB, Kent TS, Chaikof EL, Vollmer CM (2013). A prospectively validated clinical risk score accurately predicts pancreatic fistula after pancreatoduodenectomy. J Am Coll Surg.

[12] Gouillat C, Chipponi J, Baulieux J, Partensky C, Saric J, Gayet B (2001). Randomized controlled multicentre trial of somatostatin infusion after pancreaticoduodenectomy. Br J Surg.

[13] Adiamah A, Arif Z, Berti F, Singh S, Laskar N, Gomez D (2019). The use of prophylactic somatostatin therapy following pancreaticoduodenectomy: a meta-analysis of randomised controlled trials. World J Surg.

[14] Visschers RG, Olde Damink SW, Winkens B, Soeters PB, van Gemert WG (2008). Treatment strategies in 135 consecutive patients with enterocutaneous fistulas. World J Surg.

[15] Yao Z, Tian W, Huang M, Xu X, Zhao R (2022). Effect of placing double-lumen irrigation-suction tube on closure of anastomotic defect following rectal cancer surgery. Surg Endosc.

[16] Corleto VD (2010). Somatostatin and the gastrointestinal tract. Curr Opin Endocrinol Diabetes Obes.

[17] Gomes-Porras M, Cárdenas-Salas J, Álvarez-Escolá C (2020). Somatostatin analogs in clinical practice: a review. Int J Mol Sci.

[18] Coughlin S, Roth L, Lurati G, Faulhaber M (2012). Somatostatin analogues for the treatment of enterocutaneous fistulas: a systematic review and meta-analysis. World J Surg.

[19] Klempa I, Schwedes U, Usadel KH (1979). Verhütung von postoperativen pankreatitischen Komplikationen nach Duodenopankreatektomie durch Somatostatin [Prevention of postoperative pancreatic complications following duodenopancreatectomy using somatostatin]. Chirurg.

[20] Suzuki H, Yamada K, Matsuda Y, Onozuka M, Yamamoto T (2017). CXCL14-like immunoreactivity exists in somatostatin-containing endocrine cells, and in the lamina propria and submucosal somatostatinergic nervous system of mouse alimentary tract. Acta Histochem Cytochem.

[21] Shamsi BH, Chatoo M, Xu XK, Xu X, Chen XQ (2021). Versatile functions of somatostatin and somatostatin receptors in the gastrointestinal system. Front Endocrinol (Lausanne).

[22] Boelens PG, Heesakkers FF, Luyer MD (2014). Reduction of postoperative ileus by early enteral nutrition in patients undergoing major rectal surgery: prospective, randomized, controlled trial. Ann Surg.

[23] Tian W, Xu X, Yao Z (2021). Early enteral nutrition could reduce risk of recurrent leakage after definitive resection of anastomotic leakage after colorectal cancer surgery. World J Surg.

[24] Kubba AK, Dallal H, Haydon GH, Hayes PC, Palmer KR (1999). The effect of octreotide on gastroduodenal blood flow measured by laser Doppler flowmetry in rabbits and man. Am J Gastroenterol.

[25] Barrie R, Woltering EA, Hajarizadeh H, Mueller C, Ure T, Fletcher WS (1993). Inhibition of angiogenesis by somatostatin and somatostatin-like compounds is structurally dependent. J Surg Res.

[26] Yang F, Liu D, Xu X (2020). A double-lumen irrigation-suction tube placed during operation could reduce the risk of grade C anastomotic leakage resulting from selective sigmoid colon cancer radical resection. Langenbecks Arch Surg.

[27] McMillan MT, Christein JD, Callery MP (2014). Prophylactic octreotide for pancreatoduodenectomy: more harm than good?. HPB (Oxford).

